# How to Teach about What Is a Species

**DOI:** 10.3390/biology10060523

**Published:** 2021-06-11

**Authors:** Matthias Wolf

**Affiliations:** Department of Bioinformatics, Biocenter, University of Würzburg, Am Hubland, 97074 Würzburg, Germany; matthias.wolf@biozentrum.uni-wuerzburg.de

**Keywords:** biospecies, species as individuals, species as natural kinds, species concept, species problem

## Abstract

**Simple Summary:**

What is a species? According to the biospecies concept, organisms that can mate with each other belong to a species, e.g., *Homo sapiens*. But does this adequately explain to us what a species is? No! With the help of the biospecies concept, we can only recognize species, but we cannot explain them. To understand what a species is, we must ask ourselves and our students if a species is real—a thing with properties (comparable to an individual)—or just a concept, a natural class of organisms with largely equivalent characteristics.

**Abstract:**

To ask students what a species is always has something rhetorical about it. Too quickly comes the rote answer, often learned by heart without ever thinking about it: “A species is a reproductive community of populations (reproductively isolated from others), which occupies a specific niche in nature” (Mayr 1982). However, do two people look alike because they are twins or are they twins because they look alike? “Two organisms do not belong to the same species because they mate and reproduce, but they only are able to do so because they belong to the same species” (Mahner and Bunge 1997). Unfortunately, most biology (pre-university) teachers have no opinion on whether species are real or conceptual, simply because they have never been taught the question themselves, but rather one answer they still pass on to their students today, learned by heart without ever thinking about it. Species are either real or conceptual and, in my opinion, it is this “or” that we should teach about. Only then can we discuss those fundamental questions such as who or what is selected, who or what evolves and, finally, what is biodiversity and phylogenetics all about? Individuals related to each other by the tree of life.

## 1. Background

“No one definition has as yet satisfied all naturalists; yet every naturalist knows vaguely what he means when he speaks of a species.” [*On the Origin of Species* [1], Chapter II, Charles Darwin, 1859]

In biology classes at school, children learn what a species is, and from my own experience, I know that most biology students, later at university, assume they know what a species is. When asked about it, their answer usually comes like Lady Gaga sang, faster than you can say Ferrari. They come forward and say the same thing, year after year, as if a debate about it is not even conceivable. In reality, biology certainly has one of its main problems here, and I think most lecturers avoid the discussion because they themselves feel insecure and uncomfortable with it, or worse, just like their students, have only one standard answer in their repertoire. However, what is the species problem (certainly more than discussing asexual organisms and/or agamospecies), and how to teach about what is a species? There is certainly a philosophical discussion to have with students about the reality of species. We can have them face ontological questions and/or let them work out such questions themselves. The latter would be particularly instructive. Students are actually far cleverer than we give them credit for—they are not only always asking about exceptions, but also willing to discuss the philosophy of biology.

## 2. Main Text

Ontological discussions around the concept of species are as old as biology itself; yet, these discussions largely bypass our school lessons. In school, we only discuss different ways to recognize species (biospecies, ecospecies, morphospecies, and chronospecies). My viewpoint is, therefore, not meant to be another philosophical treatise on the species problem; it is only meant to make the problem briefly and concisely understandable for students and teachers. We should not be afraid to debate ontological questions with our students. In the context of a scientific education, a clear and defined language is essential. We should not use the term “species” without facing its ontology at least once.

In the minds of students, the concepts of species and evolution somehow belong together, but few know exactly how. “Evolution amounts to speciation, that is, to the emergence of qualitatively novel organisms” (Mahner and Bunge 1997) [2]. Evolution is driven by random mutations and natural selection; the latter is an individual–environment interaction. Individuals are selected (survival of the fittest) and populations (individuals with binding relations) evolve. A species is a natural kind (of equivalent organisms), but step by step: To ask students what a species is always has something rhetorical about it. The rote answer comes all too quickly, learned by heart without ever thinking about it: “A species is a reproductive community of populations (reproductively isolated from others), which occupies a specific niche in nature” (Mayr 1982) [3]. Even if several objections could be raised against this “definition”, it is maybe the best-known statement in modern biology. “In fact, it is an indicator hypothesis: it does not tell us what a biospecies is but how to recognize it, namely by observing reproduction or else by failing to observe the latter. Neither [mutual] reproduction nor [sexual] isolation are defining properties of a species but, at best, properties of organisms that may be used as symptoms of the latters’ membership in a particular species. In other words, two organisms do not belong to the same species because they mate and reproduce, but they only are able to do so because they belong to the same species” (Mahner and Bunge 1997) [2]. (I had already cited this paragraph in Müller et al. (2007) [4] in the context of an original paper (on ITS2 secondary structures and distinguishing species), but, with this viewpoint here, I would like to give my students (especially the prospective biology teachers) a more general and shorter overview (a handout) of the species problem.) I was very lucky to have had good teachers (I actually met Ernst Mayr and Martin Mahner); they asked questions, and did not necessarily provide answers: “Do two people look alike because they are twins or are they twins because they look alike?” I learned to distinguish between recognition and explanation. Biospecies, chronospecies, morphospecies, ecospecies, etc., all those concepts are merely correlated indicator hypotheses. They only enable us to recognize species. Such indicator hypotheses are not definitions and they do not tell us anything about what a species is. The latter is an ontological question and there are two competing concepts, SAI and SANK. SAI (species as individuals) considers a species as an individual carrier of properties; the species evolves. Individuals in relation to species are not elements, but are degraded to an illogical part–whole relationship. SANK (species as natural kinds) considers (and this sounds more logical to me) the individuals as carriers of properties; here, populations (i.e., individuals with binding relations) evolve. Accordingly, species—individuals with equivalent properties—are natural kinds (classes of equivalent organisms), and thus, species are conceptual. SAI- or SANK-concepts allow the definition of species; they explain species as real or conceptual! On this subject, with only a few exceptions, there is little easy to read literature for students or teachers. However, I strongly recommend the book “Multicellular Animals—A New Approach to the Phylogenetic Order in Nature—Volume I” by Peter Ax (1996) [5]; and with respect to *Homo sapiens*, species concepts are excellently discussed by Walter Sudhaus (2020) [6]. In practice, biologists are not or only rarely confronted with SAI and/or SANK, i.e., in practice biologists work with the above-mentioned indicator hypotheses. Nevertheless, the ontological question about the nature of species is, in my opinion, relevant, particularly for teaching. The biospecies concept from Ernst Mayr was considered as a definition and was accused of having confused the reason for recognition with the reason for explanation—a classical SAI/SANK debate, and I was right in the middle. Do two people look alike because they are twins or are they twins because they look alike? Two organisms do not belong to the same species because they mate and reproduce, but they only are able to do so because they belong to the same species. What is a species? Sometimes teaching questions enlightens students more than giving them answers. Most biology teachers unfortunately have no opinion on whether species are real or conceptual, simply because they have never been taught the question themselves, but rather one answer they still pass on to their students today, learned by heart without ever thinking about it. How to teach about what is a species? We should teach about what a definition is, about cladistics, about what is real (what is reality) and what is science all about—questions and not just answers learned by rote. Teach problem awareness, follow up questions and the consequences of answers. The “species” concept in school science (including a literature survey) has been extensively reviewed by Nyléhn und Ødegaard (2018) [7]; however, an ontological debate plays only a subordinate role here. From a pedagogical point of view, do not be afraid to venture into a philosophical excursion in your biology class. Use scenarios, associations, small group activities and context clues. Put the term “species” into context and/or create a story around the term “species”. Instruct your students to write sentences using the term “species” and/or read sentences with the word “species” embedded, e.g., talk about “species” extinction, about invasive “species”, about distinguishing “species”, etc. As a philosophical exercise, use the twin example as described above and/or just ask your students who can swim, dance, laugh, or cry? Can a “species” do anything at all? Is it possible to examine a “species”? Do we need at all the term “species”, or do we need even more terms for the different meanings of the term? Concerning the latter, for further reading and for an in-depth discussion of natural kinds *sensu lato*, *sensu stricto*, of more than 30 different species concepts (including ontological and operational species concepts), see Mahner and Bunge (1997) [2] and/or Zachos (2015) [8]. Therein also, the historical development, as well as the practical relevance of different species concepts, is thoroughly discussed. The discussion about what a species is will certainly continue. Arguments against my viewpoint you may want to find e.g., in “The reality of species: real phenomena not theoretical objects” by Wilkins (2017) [9]. I would like to end fairly with one of his questions, “What is a mountain?” and last but not least with a final statement by myself: Not species die but individuals and each is biodiversity worth protecting, the latter I hope we can all agree with. 

## 3. Conclusions

How to teach about what is a species? Species are either real or conceptual and, in my opinion, it is this “or” that we should teach about. Only then we can discuss those fundamental questions such as who or what is selected, who or what evolves and, finally, what is biodiversity and phylogenetics all about? Individuals related to each other by the tree of life! In my opinion, there are populations—real individuals with binding relations—which we can summarize under a species name. Species are formed in the mind of the investigator (cf. Sudhaus (2020) [6]): “A species is a *biospecies* if, and only if, it is a natural kind” (Mahner and Bunge (1997) [2]). A biological species is a natural class of organisms with largely equivalent characteristics. Complementing this, Frank Zachos (2016) wrote, “What is important to note is that when species are viewed as classes with essential properties, this does not necessarily entail that they are unnatural and completely arbitrary groupings.” Actually, Zachos quoted Bird and Tobin (2015): “To say that a kind is natural is to say that it corresponds to a grouping that reflects the structure of the natural world rather than the interests and actions of human beings. We tend to assume that science is often successful in revealing these kinds; it is a corollary of scientific realism that when all goes well the classifications and taxonomies employed by science correspond to the real kinds in nature. The existence of these real and independent kinds of things is held to justify our scientific inferences and practices” [8,10].

## Data Availability

Not applicable.
